# Relationship between Nutritional Support and Tuberculosis Treatment Outcomes in West Bengal, India

**DOI:** 10.4236/jtr.2016.44023

**Published:** 2016-12-21

**Authors:** Blesson Samuel, Tyson Volkmann, Sushma Cornelius, Sugata Mukhopadhay, MejoJose, Kaushik Mitra, Ajay M. V. Kumar, John E. Oeltmann, Sidhajyoti Parija, Aslesh Ottapura Prabhakaran, Patrick K. Moonan, Vineet K. Chadha

**Affiliations:** 1 World Vision India, New Delhi, India; 2 Centers for Disease Control and Prevention, Atlanta, USA; 3 Burdwan Medical College, West Bengal, India; 4 International Union against Tuberculosis and Lung Disease, South-East Asia Regional Office, New Delhi, India; 5 Southern Health Improvement Samity, West Bengal, India; 6 Department of Community Medicine, Academy of Medical Sciences, Pariyaram, India; 7 Epidemiology and Research Division, National Tuberculosis Institute, Bangalore, India

**Keywords:** Nutritional Support, Poverty, Pulmonary Tuberculosis, India

## Abstract

**Introduction:**

Poverty and poor nutrition are associated with the risk of developing tuberculosis (TB). Socioeconomic factors may interfere with anti-tuberculosis treatment compliance and its outcome. We examined whether providing nutritional support (monthly supply of rice and lentil beans) to TB patients who live below the poverty line was associated with TB treatment outcome.

**Methods:**

This was a retrospective cohort study of sputum smear-positive pulmonary TB patients living below the poverty line (income of <$1.25 per day) registered for anti-tuberculosis treatment in two rural districts of West Bengal, India during 2012 to 2013. We compared treatment outcomes among patients who received nutritional support with those who did not. A log-binomial regression model was used to assess the relation between nutritional support and unsuccessful treatment outcome (loss-to-follow-up, treatment failure and death).

**Results:**

Of 173 TB patients provided nutritional support, 15 (9%) had unsuccessful treatment outcomes, while 84 (21%) of the 400 not provided nutrition support had unsuccessful treatment outcomes (p < 0.001). After adjusting for age, sex and previous treatment, those who received nutritional support had a 50% reduced risk of unsuccessful treatment outcome than those who did not receive nutritional support (Relative Risk: 0.51; 95% Confidence Intervals: 0.30 - 0.86).

**Conclusion:**

Under programmatic conditions, monthly rations of rice and lentils were associated with lower risk of unsuccessful treatment outcome among impoverished TB patients. Given the relatively small financial commitment needed per patient ($10 per patient per month), the national TB programme should consider scaling up nutritional support among TB patients living below the poverty line.

## 1. Introduction

India has the highest tuberculosis (TB) incidence in the world in terms of absolute numbers. In 2014, about 2 million people in India are estimated to have developed TB disease—22% of the 9 million TB cases worldwide [1]. Poverty has been long associated both with under-nutrition and TB [2] [3] [4]. Lack of access to basic health services, food insecurity and inadequate living conditions fuels TB transmission. A vicious relationship exists between poverty and TB; the impoverished are at greater risk of TB, and those with TB are at greater risk of becoming impoverished [2] [4]. Studies suggest that the average TB patient loses three to four months of individual and household income due to incapacitation and while seeking diagnosis and treatment [5] [6]. For people living below poverty line (BPL) (<US$1.25 per day), this could mean the difference between feeding themselves and their families or complying with anti-tuberculosis treatment—a minimum course of at least 6 months. Nutrition plays an important role in general well-being but also healing from infections like *M. tuberculosis* [7] [8]. Unfortunately, the impoverished suffer from high rates of under-nutrition, which besides being risk factor for development of TB disease, can also adversely affect outcomes of TB treatment [9] [10] [11].

In studies from India, the impoverished were twice as likely to have TB, three times less likely to access TB care, four times less likely to complete anti-tuberculosis treatment, and more likely to incur impoverishing payments for TB care than the non-impoverished [12] [13]. The risk of mortality during anti-tuberculosis treatment among severely undernourished patients was double that of non-undernourished patients in rural and central India [14].

While provision of free food has been shown to increase weight during treatment and improve quality of life, there is conflicting evidence if it improves TB treatment outcomes, with some trials showing benefit and others not [14] [15] [16] [17]. Hence, we aimed to determine if, among the poor, nutritional support was associated with treatment outcome among pulmonary TB patients, in select TB units in West Bengal, India.

## 2. Methods

### 2.1. Setting

The study was conducted in two districts in West Bengal, India—South 24 Parganas and North 24 Parganas having populations of 8.2 million and 10.1 million respectively, as per the District census in 2011 [18]. About 82% of the households in rural North 24 Parganas suffered from food insecurity and 32% of the population lived below the poverty line [19]. In South 24 Parganas, 35% of the population lived below the poverty line. [20].

In two tuberculosis units (TUs), one in each district, a non-profit organization called Southern Health Improvement Samitee (SHIS) provided monthly allotments of rice (13 kilograms) and lentil beans (3 kilograms) at no cost to TB patients for a period ranging from 60 to 90 days. A line-list of patient names from SHIS was used to determine who received nutritional support and who did not. No other organization provided any nutritional support to the selected TB patients during the study period, nor was any free nutrition provided to any TB patients during treatment by the government.

### 2.2. Study Design, Study Population and Study Period

This was a retrospective cohort study of newly diagnosed and previously treated sputum smear-positive, pulmonary TB patients living below the poverty line registered for anti-tuberculosis treatment in four tuberculosis units during 2012-2013. Patients with monthly income below 3000 INR (<US$46) were considered living below the poverty line, or impoverished. Patients with extra-pulmonary disease, multidrug-resistant tuberculosis, or with monthly incomes above 3000 INR were excluded from analysis.

#### Given Nutrition support group

In the two TUs where nutritional support was being implemented, a line list of patients who actually received the support was obtained from SHIS.

#### Not given Nutrition support group

A sample of TB patients (*i.e.*, every second patient) that lived below the poverty line from two TUs of the districts who did not receive free food support was considered the non-intervention group. Non-intervention TUs were selected due to their location near to the intervention TUs. In total there are 17 TUs in South 24 Praganas and 21 TUs in the District North 24 Praganas. To increase the power of the study, twice as many patients in the non-intervention group as compared to intervention group were selected for enrolment. A line listing of 400 BPL TB patients with an income of below INR 3000 per month and who had not received nutrition was validated through senior treatment supervisors (STS). The estimated sample size was attained by including all patients registered under RNTCP at these facilities during 2012-2013.

#### Outcome

Treatment outcomes were recorded for all patients from the national TB programme registers. Completing six months of anti-tuberculosis treatment with or without having at least two sputum smear-negative test results by month six or eight of treatment were considered successful outcomes. Loss-to-follow-up (*i.e.*, missing two months or more of anti-tuberculosis treatment consecutively), treatment failure (*i.e.*, sputum smear-positive test results at month 5 or more after starting anti-tuberculosis treatment), or death during anti-tuberculosis treatment was considered unsuccessful outcomes.

#### Study variables and sources of data

The following patient characteristics were extracted from the national TB programme registers to be used in this analysis: sex, age, year registered, treatment outcome, type of patient and treatment outcome.

### 2.3. Statistical Methods

We compared differences in the proportion of patient with unsuccessful treatment outcome by nutritional support, sex, age group, Type of TB patient (newly diagnosed, previously treated), year registered, using chi-square test. A p value of <0.05 was considered statistically significant. A log-binomial regression model was used to calculate adjusted relative risk (RR) and 95% confidence intervals (CI) to find out the association between nutritional support and unsuccessful treatment outcome if any.

## 3. Results

In the two TUs where nutrition support was provided, a total of 737 TB patients living below the poverty line were registered for anti-tuberculosis treatment during 2012-2013. Among the 315 BPL patients who received nutritional support, 142 (45%) had extra-pulmonary disease or MDR-TB and were excluded. Thus, a total of 173 pulmonary TB patients who received nutritional support and anti-TB treatment under RNTCP with first line drugs were included in analysis. In the TUs not implementing nutritional support, 1062 TB patients living below the poverty line were registered for anti-tuberculosis treatment during 2012-2013. A total of 400 pulmonary, non-MDR TB patients were selected.

Of the 573 patients included in the analysis, 159 (28%) were female and 417 (72%) were male (Table 1) and 347 (61%) were less than 45 years old. There were 479 (84%) new cases and 94 (16%) retreatment cases. The baseline characteristics of intervention and non-intervention group were similar by age, sex, history of treatment (Table 1).

Of the 173 TB patients who were supported by nutrition, 15 (9%) had unsuccessful treatment outcomes, and 84 (21%) of those who were not provided nutrition support had unsuccessful treatment outcomes (p < 0.001) (RR: 0.41; 95% CI: 0.21 – 0.60).

After adjusting for age, sex, and previous treatment, those receiving nutritional support had nearly a 50% lesser risk of unsuccessful treatment outcome than those who did not receive nutritional support (RR: 0.51; 95% CI: 0.30 – 0.86).

## 4. Discussion

This study found that food support for patients living below the poverty line was associated with lower risk of unsuccessful TB treatment outcomes. While some studies have asserted that food support interventions might have the potential to improve adherence to TB treatment, evidence concerning the effect of nutritional support on TB outcomes is sparse. In our study, the simple provision of US$10 of rice and lentil beans provided once a month was associated with a significantly lower risk of unsuccessful treatment outcomes, even after adjusting for known co-factors.

The results should be interpreted with caution given the inherent limitations of an observational study. While the intervention and non-intervention groups were similar with respect to age, sex and previous history of treatment, we do not have information about other possible co-factors (HIV status, diabetes mellitus, tobacco smoking, alcohol intake, baseline nutritional status, and disease severity). The classification of living below the poverty line was based on self-reported income data. We were unable to conduct multivariate analysis on the disaggregated treatment outcomes because small numbers of patients receiving nutrition died, were lost to follow up, or failed to treat. Another limitation relates to lack of information on long-term treatment outcomes like relapse given the reliance of the study on routinely collected data. We plan to follow these cohorts to see if improved treatment success at the end of treatment translates to better long-term outcomes.

The evidence that food supplied to poor TB patients might be associated with greater treatment success could be vital for policy makers to make adequate provision for need-based nutrition schemes easily accessible to poor patients across India. Finally, it is important to note that this intervention costs as little as $10 per month, a small cost relative to that of unsuccessful treatment outcomes. Further studies should explore this effect prospectively using a randomized control trial design with adequate sample size.

## Figures and Tables

**Table 1:** Baseline characteristics of BPL TB patients (stratified by nutritional support) registered in West Bengal, India, 2012–2013.

Characteristic	Nutritional support (%)	No nutritional support (%)	Total (%)	P-value
**Total**	173 (30)	400 (70)	573 (100)	--
**Sex**				
Female	50 (29)	106 (27)	159 (27)	0.55
Male	123 (71)	294 (74)	417 (73)	
**Age (in years)**				
<15	2 (1)	4 (1)	6 (1)	0.99
15 – 44	102 (59)	239 (60)	341 (60)	
45 – 64	51 (30)	117 (29)	168 (29)	
65+	18 (10)	40 (10)	58 (10)	
**TB classification**				
New	141 (82)	338 (85)	479 (84)	0.37
Previously treated	32 (19)	62 (16)	94 (16)	
**Treatment outcome (combined)**				
Successful	158 (91)	316 (79)	99 (17)	<0.001
Unsuccessful	15 (9)	84 (21)	474 (83)	
**Treatment Outcome (disaggregated)**				
Cure	130 (75)	220 (55)	350 (61)	<0.001
Treatment completed	28 (16)	96 (24)	124 (22)	
Lost to follow up	1 (1)	41 (10)	42 (7)	
Failure	6 (4)	10 (3)	16 (3)	
Death	8 (5)	33 (8)	41 (7)	
